# Development and formative evaluation of website‐based tool to support healthy lifestyles in family day care

**DOI:** 10.1002/hpja.947

**Published:** 2025-01-08

**Authors:** Georgie Tran, Bridget Kelly, Sarah T. Ryan, Megan Hammersley, Erin Kerr, Jennifer Norman, Mel Leedham, Cecilia Vuong, Karen Wardle, Kainaat Syed, Anthony Okely

**Affiliations:** ^1^ School of Health and Society, Faculty of the Arts, Social Sciences and Humanities University of Wollongong Wollongong New South Wales Australia; ^2^ Health Promotion Service Sydney Local Health District Camperdown New South Wales Australia; ^3^ Health Promotion Service Illawarra Shoalhaven Local Health District Warrawong New South Wales Australia; ^4^ Health Promotion Service South Western Sydney Local Health District Liverpool New South Wales Australia

**Keywords:** family child care home, family day care, health education, nutrition, physical activity, professional development, quality improvement

## Abstract

**Issue Addressed:**

Nutrition and physical activity practices in Australian family day care are suboptimal. A web‐based tool was co‐developed with family day care service providers and educators, health promotion staff and the New South Wales Ministry of Health to promote healthier nutrition and physical activity practices through an existing quality improvement process.

**Methods:**

Formative evaluation was conducted in January–February 2023. An online survey included 13 questions relating to content, language, structure, ease of usage and participant satisfaction. Questions used Likert scales to rate participants' experience from poor to excellent. Tool usage data were extracted from the website to reflect user activity. Service providers (*n* = 3) and educators (*n* = 9) tested the tool for 4 weeks.

**Results:**

Survey questions were grouped into measures of perceived convenience, difficulty and helpfulness. All participants chose a rating of ‘good’ or ‘excellent’ across all questions, with a higher proportion of participants rating the questions as ‘excellent’. All participants used the tool at least once. Perceived value of research was the main theme that emerged from the open text feedback.

**Conclusions:**

Results suggest that the tool was feasible, easy to use and relevant to practice.

**So What?:**

This is the first known tool designed for family day care to promote healthier nutrition and physical activity practices through an existing quality improvement process to implement change. An efficacy trial will follow to evaluate effectiveness. The tool is predicted to serve as a platform for identifying gaps between policy and practice and for facilitating practice improvements.

## 1 INTRODUCTION

Early childhood education and care settings, including family day care (FDC) in Australia, play an essential role in fostering healthy habits in young children.^1^ Overseas equivalents include ‘home‐based care’ in New Zealand and ‘family child care’ in Canada and United States.^2^ FDC involves educators providing care from their homes. They are registered with a service provider who must ensure their educators are appropriately trained and supported. With correct training and support, educators can influence healthy behaviours in children.^3^

Quality improvement in FDC involves a mandatory Quality Improvement Plan (QIP) as part of Government registration. The QIP must be reviewed at least annually, with consideration to the National Quality Standard (NQS).^4^ There is also guidance from initiatives such as *Munch & Move*, a program available to all child care services in New South Wales (NSW) Australia. It encourages and supports services to implement healthy eating and physical activity strategies for children in their care.^5^

The sector has identified the need for a tool to promote healthy practices and monitor such activity by improving understanding of *Munch & Move* program elements and generating implementation evidence.^6^ No tool exists in FDC to improve nutrition and physical activity practices and online platforms can be a practical method to assist in quality improvement.^7^ This study developed and conducted a formative evaluation to assess the perceived usability of a website‐based tool for service providers and educators to promote healthier nutrition and physical activity practices.

## 2 METHODS

This study followed the Standards for Quality Improvement Reporting Excellence in Education guidelines (SQUIRE‐EDU) and received approval from the University of Wollongong Human Research Ethics Committee (2022/PID01883). Online informed consent was obtained from all participants.

### 2.1 Tool development

Resources from *Munch & Move* were used to help guide development. Online focus groups were conducted to obtain stakeholder input. A review of the literature was conducted to determine the requirements of a QIP review. A working group consisting of the academic research team and key health promotion staff from Local Health Districts (LHDs) and NSW Ministry of Health met monthly for content and design discussions of the tool. An advisory panel of three educators provided early‐stage feedback.

### 2.2 Tool description

The *Family Day Care Quality Improvement Toolkit* (the Toolkit), was hosted on a website with user login functionality and written in English. Service providers and educators accessed the same Toolkit, however accessible features differed.

For educators, the Toolkit contained a self‐assessment questionnaire which was developed based on *Munch & Move* internal documents from NSW Ministry of Health. The questionnaire linked with relevant NQS and *Munch & Move* key messages and each question had a response option rating out of 3 (0 = lowest score, 3 = highest score). A score of 0 indicated that the *Munch & Move* practice was not being implemented at all. A score of 1 indicated limited implementation, 2 indicated partial implementation and 3 indicated full implementation. If an educator did not receive the highest rating, targeted educational *Munch & Move* resources were provided. There was also a self‐assessment reflection component to guide critical reflection.

For service providers, there was a tailored dashboard with visual and written summaries of their educators' performance, highlighting priority areas related to *Munch & Move* practices. Service providers could then complete their required QIP review with the option of utilising the ‘Action Plan’ template, which was adapted from the Australian Children's Education and Care Quality Authority QIP template.^4^ Table 1 shows an overview of the Toolkit content.

**TABLE 1:** Overview of the flow of content in the *Family Day Care Quality Improvement Toolkit*.

Section title	Content
Service provider account	
Step 1	View educator self‐assessment questionnaires——users were able to view individual educator results from completed questionnaires.
Step 2	View educator self‐assessment reflections—users were able to view individual educator reflections that had been submitted.
Step 3	Prioritise areas for improvement—nutrition and physical activity priority areas were presented to the user based on the results of the educators' self‐assessment questionnaires (from Step 1). The user also had the option to select a tick‐box stating that they consulted with educators offline and could manually override the automatic selection of priority areas if they felt that it did not accurately reflect what their priority areas were.
Step 4	Complete Action/Improvement Plan (optional)—contained prompts and guides to complete a detailed action plan based on the priority improvement areas identified in Step 3. The completed action plan could be downloaded as an editable Microsoft Word document for easy incorporation into a service's QIP.
All action plans	Collation of all completed action plans that were submitted by the service provider (from Step 4).
Dashboard (reports)	Overview reports were created, which contained visual and written summaries of all educators' self‐reflections and questionnaires, and highlighted key priority areas related to *Munch & Move* practices using a traffic light system for easy visualisation.
Educator account	
Step 1	Complete a new self‐assessment questionnaire—designed to directly link with the relevant NQS and *Munch & Move* key messages, and each question given a rating out of 3 (0 = lowest score, 3 = highest score).
Step 2	Complete a new self‐assessment reflection—assists in linking reflections directly to the NQS. This step automatically followed Step 1 to allow for reflection on the identified priority areas.
Step 3	View previous questionnaire results and tailored resources—educators could view their results and their performance based on the NQS and *Munch & Move* key messages. If an educator did not receive the highest rating (3/3) for a question, targeted educational resources were provided.

### 2.3 Data collection

A convenience sample of service providers already supported by the health promotion teams from Illawarra Shoalhaven LHD and South Western Sydney LHD were recruited. There was a target sample of five service providers. Service providers were asked to involve their educators, as per normal processes of reviewing a QIP. Those not undergoing a QIP review were excluded.

Data were collected between January and February 2023. Participants were provided with access to the Toolkit over 4 weeks. Participants accessed the Toolkit for 4 weeks and completed an online survey (REDCap version 14.0.10) for demographic and feasibility data. Data were analysed using descriptive statistics and thematic analysis (NVivo version 12.7).

The survey questions used a 5‐point Likert scale. An optional free‐text response was included. The questions related to the Toolkit's: perceived convenience; functionality; usefulness, and; their understanding of the information and interest in using the Toolkit again. Tool usage data was extracted on user's activity and included: uptake of Toolkit (number of participants who recorded at least one activity) and complete usage of Toolkit (number of participants who utilised all features).

## 3 RESULTS

Ten service providers expressed interest, of which three completed the study and were included in the final analysis. Nine educators completed the study, contributing to a total of 12 participants. Of the seven service providers who did not complete the study, three had no educators interested in participating, two had already reviewed their QIP prior to the study and two withdrew their interest.

All participants held a Certificate III or Diploma to work in the sector. Most (11/12) participants had worked more than 2 years in their service.

All participants recorded at least one activity in the Toolkit. Most (9/12) participants utilised all features or recorded at least one activity in all features/steps of the Toolkit. All service providers utilised the optional ‘Action Plan’ template.

Table 2 summarises the user feedback on the Toolkit. All participants completed the post‐study survey. All participants chose a rating of ‘good’ or ‘excellent’ across all feasibility questions.

**TABLE 2:** Feasibility outcomes self‐rated by participants using a 5‐point Likert scale (*n* = 12).

		Frequency of ratings *n*				
Measure	Question	1—Very poor	2—Poor	3—Fair	4—Good	5—Excellent
Perceived convenience	How would you rate being able to access the website?	0	0	0	5	7
Perceived difficulty	How would you rate the functionality of the website (i.e., logging in, navigating the features)?	0	0	0	4	8
	How would you rate your understanding of the information on the website?	0	0	0	5	7
Perceived helpfulness	How would you rate the usefulness of the website overall?	0	0	0	4	8
	How would you rate your interest level in using the website for quality improvement?	0	0	0	4	8

Educators and service providers were asked additional questions regarding features specifically available to them. Seven of nine educators rated the usefulness of the questionnaire as excellent and two of nine rated it as good. All educators responded that the length of the questionnaire was just right. Most educators (6/9) rated the usefulness of the educational resources as excellent. Similarly, six of nine rated the relevance of the self‐assessment reflection feature as excellent. For service providers, two of three rated the usefulness of the Action Plan as excellent and one of three rated it as good. All three service providers stated that the Action Plan was easy to use.

The open text feedback was completed by three participants. One main theme emerged: perceived value of research. Most participants (2/3) expressed their support for the research project, noting the importance of the research.

## 4 DISCUSSION

The Toolkit was well received, with positive ratings across all feasibility questions. All participants used the Toolkit at least once, with most using all features of the Toolkit. Based on a small sample, service providers offered positive feedback regarding the perceived value of the research project, ease of tool use and relevance of the Toolkit in practice. The Toolkit's design uniquely integrates health promotion action into an existing quality improvement process, leveraging the annual QIP review to help service providers implement healthier practices.

The positive ratings across the feasibility questions in the present study indicated that the Toolkit was perceived as convenient and easy to use, and this may highlight the benefits of using a digital platform. Similarly, an Australian pilot study of a digital tool designed to improve reporting of pastoral care activities indicated that three‐quarters of users found the digital tool more convenient than the traditional paper‐based method.^8^ Another study that tested the use of a digital tool designed for professionals in social, health and education settings to promote the health and wellbeing of children found that the tool was considered to be easy to use and made it simple for professionals to explain to children about healthy behaviours.^9^ In the present study, it is likely that the Toolkit's features may have simplified the quality improvement planning process and allowed easy access to all required data on a single website platform.

The positive response to the Toolkit may also be attributed to the co‐development and consultation with the sector during development. This highlights the importance of the partnership process. A Danish study examined partnership synergy in health promotion and found three factors that must be considered in shared decision‐making, including having a common purpose, aligning all partners' self‐interests, and enabling action. This allows collaborators to recognise their interdependencies.^10^ For example, the focus groups/interview conducted in the present study, along with the advisory panel of educators, allowed the research team to understand sector needs and professional priorities. In addition, leveraging on existing relationships with health promotion staff who have implemented *Munch & Move* in their LHDs could provide assistance in recruitment and advise on any barriers that arose in the present study.

Based on the Toolkit's positive ratings in perceived helpfulness in the present study, along with the responses to the open‐ended question, service providers also perceived that the Toolkit was relevant to practice. An earlier randomised controlled trial of a website‐based teaching resource for medical professionals to improve quality of child development assessment found that the digital resource significantly improved knowledge, increased confidence and was found to be useful to practice.^11^ Another study examining the effectiveness of a digital tool developed to support secondary teachers' professional development found that the quality of teachers' self‐assessment of their classes significantly improved after using the digital tool.^12^ This indicates that users are likely to perceive a tool as relevant to practice if it assists in their professional development or initiates quality improvement. In the present study, the Toolkit assisted service providers with the annual QIP review and guided educators through their critical reflection. For service providers, this included a tailored dashboard summarising educators' performance on *Munch & Move* practices, identifying key areas for improvement and a structured Action Plan template to set measurable objectives and integrate health promotion within broader quality improvement goals. For educators, the Toolkit evaluated adherence to *Munch & Move* practices and engaged them in critical reflection. Hence, participants in the present study perceived the Toolkit as relevant to practice.

Reflective practice is a key contributor to professional development, skills maintenance and establishment of good practice. By actively evaluating current practices, professionals can gain a new perspective which drives the process of quality improvement.^13^ A systematic review exploring how quality improvement collaboratives can lead to better outcomes found that participation in quality improvement projects may improve knowledge, skills, attitude and shared leadership.^14^ In the present study, the results suggest that the participants found the Toolkit useful to practice and this may be largely due to the Toolkit assisting with quality improvement.

Limitations include the small sample size and therefore results are not generalisable. There was no control condition or efficacy measurements and conclusions can only be drawn on feasibility. Two service providers withdrew their interest and it is likely that the remaining participants were the most engaged in the research topic.

## 5 CONCLUSION

To our knowledge, this is the first tool in FDC to promote healthier nutrition and physical activity practices by using an existing quality improvement process to implement change. The tool is predicted to serve as a platform for identifying gaps between policy and practice and for facilitating practice improvements. There are several opportunities for the tool to be embedded in practice, including delivery of the tool as part of support programs or training.

## AUTHOR CONTRIBUTIONS

All authors conceived the study and were involved in tool conceptualisation and development. GT, ML, JN, CV and KS were involved in recruitment. GT conducted data analysis and wrote the first draft of the manuscript. BK and AO provided guidance for preparing the first draft of the manuscript. All authors were involved in interpretation of data, edited the manuscript and approved the final version of the manuscript.

## CONFLICT OF INTEREST STATEMENT

The University of Wollongong collaborated with family day care service providers and educators, health promotion staff and the New South Wales Ministry of Health to develop the Toolkit.

## ETHICS STATEMENT

This study was conducted according to the guidelines laid down in the Declaration of Helsinki, and all procedures involving research participants were approved by the University of Wollongong Human Research Ethics Committee (2022/PID01883). Online informed consent was obtained from all family day care service providers and educators.

## Data Availability

The data that support the findings of this study are available from the corresponding author upon reasonable request.
